# Psychotherapeutic Interventions and Psychosocial Outcomes Following Perinatal Loss: An Umbrella Review with a Patient-Centered Care Perspective

**DOI:** 10.3390/healthcare14142141

**Published:** 2026-07-16

**Authors:** Thalia Bellali, Anna Papadopoulou, Polyxeni Liamopoulou, Chrysovalantis Karagkounis

**Affiliations:** 1 Nursing Department, International Hellenic University, Sindos, 57400 Thessaloniki, Greece; tzeni_liam@ihu.gr; 2 Department of Health Sciences, European University Cyprus, Engomi, Nicosia 1516, Cyprus; 3 Faculty of Health Sciences, School of Medicine, Aristotle University of Thessaloniki Campus, 54124 Thessaloniki, Greece; annapapadopouloupsy@gmail.com; 4 Department of Care for the Elderly, Directorate of Social Policy and Public Health, Municipality of Katerini, 60133 Katerini, Greece; kchrysov@gmail.com

**Keywords:** anxiety, depression, grief, patient-centered care, patient engagement, perinatal loss, psychosocial support, psychosocial well-being, psychotherapeutic interventions, therapeutic alliance, umbrella review

## Abstract

**Highlights:**

**What are the main findings?**

- Psychotherapeutic interventions are generally associated with improvements in grief, depression, anxiety, and post-traumatic stress symptoms following perinatal loss.
- Accessible, individualized, and technology-assisted psychological support may represent promising components of patient-centered bereavement care for bereaved parents and families.

**What are the implications of the main findings?**

- A patient-centered care perspective may help interpret how psychotherapeutic interventions support psychological adaptation after perinatal loss.
- Integrating accessible, individualized, and technology-assisted psychological support into routine bereavement care may improve psychosocial support for bereaved parents and families.

**Abstract:**

Background/Objectives: Perinatal loss is a profoundly distressing life event associated with grief, depression, anxiety, post-traumatic stress symptoms, and long-term psychosocial challenges among bereaved parents who experience miscarriage, stillbirth, or neonatal death. Although psychotherapeutic interventions are increasingly used to address these adverse outcomes, there is limited synthesis on how characteristics consistent with a patient-centered care perspective are reflected in such interventions and how they may relate to psychosocial well-being. This umbrella review aimed to synthesize evidence on psychotherapeutic interventions following perinatal loss and to examine patient-centered care–related dimensions reported across the included reviews, including therapeutic communication, patient engagement, therapeutic relationships, emotional validation, and meaning-making processes. Methods: An umbrella review was conducted in accordance with the Joanna Briggs Institute methodological guidance. Systematic reviews and meta-analyses published between 2019 and 2025 were identified through searches of PubMed, CINAHL, PsycINFO, and the Cochrane Library from database inception to 31 May 2026. Eligible reviews examined psychotherapeutic, psychosocial, and psychological support interventions designed to improve grief, depression, anxiety, post-traumatic stress symptoms, psychological distress, coping, and psychosocial well-being among bereaved parents following perinatal loss. In accordance with the predefined secondary exploratory objective, a secondary interpretive synthesis examined patient-centered care–related dimensions described within the included reviews. Results: Five systematic reviews and meta-analyses met the inclusion criteria. Interventions included cognitive behavioral therapy, mindfulness-based approaches, bereavement counseling, psychosocial support programs, narrative interventions, supportive counseling, and digitally delivered psychological therapies. Across reviews, psychotherapeutic interventions were generally associated with beneficial effects on grief, depression, anxiety, post-traumatic stress symptoms, and broader indicators of psychosocial well-being. Communication-, support-, and engagement-related characteristics consistent with a patient-centered care perspective, including empathy, therapeutic alliance, individualized support, emotional validation, and continuity of communication, were identified through secondary interpretive synthesis as recurring features of beneficial interventions. Digital modalities, such as internet-based cognitive behavioral therapy and telephone-delivered counseling, were consistently described as supporting accessibility, engagement, and continuity of care. Conclusions: Psychotherapeutic interventions following perinatal loss appear to improve a range of psychosocial outcomes. A patient-centered care perspective may help interpret how communication, emotional validation, patient engagement, and supportive therapeutic relationships are described in relation to psychological adaptation after loss. These dimensions should be understood as interpretive characteristics identified across the included reviews rather than as directly measured mechanisms of intervention effectiveness. Future research should examine communication processes, therapeutic alliance, and patient engagement using validated measures, assess how these factors relate to intervention effectiveness, and support the development of integrated, patient-centered models of perinatal bereavement care.

## 1. Introduction

Perinatal loss, encompassing miscarriage, stillbirth, and neonatal death occurring during pregnancy or within the first 28 days after birth, represents a significant global health and psychosocial challenge [1,2]. Beyond its clinical implications, perinatal loss is recognized as a profoundly distressing life event associated with substantial psychological, emotional, and social consequences for women, their partners, and families [3,4]. Evidence consistently demonstrates that individuals experiencing perinatal loss are at increased risk of anxiety, depression, post-traumatic stress symptoms, and prolonged grief reactions, which may persist for months or even years following the loss [5,6,7,8].

The impact of perinatal loss extends beyond the physical event itself and frequently challenges fundamental assumptions regarding parenthood, identity, future expectations, and family life. During pregnancy, parents often develop emotional bonds with their unborn child while simultaneously constructing hopes and expectations regarding the future. The unexpected disruption of these expectations may result in feelings of helplessness, guilt, isolation, uncertainty, and emotional suffering [9,10]. Furthermore, societal misconceptions regarding pregnancy loss and the limited public recognition of perinatal grief may contribute to the disenfranchisement of bereaved parents, leaving many individuals feeling unsupported, misunderstood, and socially isolated throughout the grieving process [11,12].

Given the substantial psychological burden associated with perinatal loss, increasing attention has been directed toward the development and implementation of psychotherapeutic and psychosocial interventions aimed at supporting bereaved parents and reducing adverse mental health outcomes. Previous studies have investigated a broad range of interventions, including cognitive behavioral therapy, mindfulness-based interventions, bereavement counseling, interpersonal counseling, supportive psychotherapy, narrative-based approaches, and digitally delivered psychological support programs [13,14,15,16,17,18,19,20,21]. Existing evidence suggests that these interventions may contribute to reductions in grief severity, depressive symptoms, anxiety, post-traumatic stress symptoms, and psychological distress following pregnancy loss or stillbirth [22,23].

While the effectiveness of psychotherapeutic interventions following perinatal loss has been examined in previous systematic reviews and meta-analyses [22,23], considerably less attention has been devoted to understanding the characteristics of interventions that may support beneficial psychosocial effects. Understanding not only whether an intervention works, but also which communication-, support-, and engagement-related characteristics are reflected in these interventions may help inform the development of more responsive psychological support for vulnerable populations [24].

Contemporary healthcare increasingly emphasizes patient-centered care as a fundamental principle for improving health outcomes and promoting psychosocial well-being [25,26]. Patient-centered care involves recognizing individuals’ values, preferences, experiences, and needs while actively involving them in decisions and processes related to their care [25]. Within this framework, effective health communication, meaningful patient engagement, therapeutic relationships, and individualized support are considered essential components of high-quality healthcare delivery and patient-centered care [27,28]. Communication enables patients to express emotions, share concerns, construct meaning from difficult experiences, and develop trusting therapeutic relationships with healthcare professionals [27]. Simultaneously, patient engagement encourages active participation in care processes, enhances self-management capabilities, promotes resilience, and fosters empowerment during periods of vulnerability and emotional distress [28].

The relevance of these concepts may be particularly important in the context of perinatal loss. Psychotherapeutic interventions are inherently communication-based processes in which therapeutic support is often described in terms of interpersonal interactions, empathy, the therapeutic alliance, collaborative goal setting, and opportunities for active engagement [12,29,30]. Therapeutic alliance has consistently been identified as one of the strongest predictors of successful psychological treatment outcomes across a wide range of mental health conditions [30]. Narrative and meaning-oriented interventions facilitate emotional processing through storytelling, reflection, and reconstruction of loss-related experiences [20,31]. In contrast, cognitive behavioral approaches typically require active participation through structured exercises, cognitive reframing, and self-reflection [13,18,19,21]. Similarly, counseling-based interventions rely heavily on supportive communication, emotional validation, continuity of care, and the establishment of trusting therapeutic relationships [14,15,16,17]. Moreover, internet-based and digitally delivered interventions may facilitate accessibility, continuity, and sustained engagement among bereaved parents who might otherwise encounter practical, geographical, or emotional barriers to receiving psychological support [18,22].

Despite growing recognition of the importance of patient-centered care in healthcare, few syntheses have examined psychotherapeutic interventions following perinatal loss from a patient-centered perspective. Existing reviews have primarily focused on intervention effectiveness and symptom reduction [22,23]. Comparatively less attention has been devoted to exploring how communication processes, therapeutic relationships, patient engagement mechanisms, emotional validation, and individualized support are reflected in intervention characteristics and how these features may relate to psychosocial outcomes. A clearer understanding of these patient-centered care–related dimensions may offer valuable insights for healthcare professionals seeking to develop and implement more responsive, compassionate, and patient-centered models of care for bereaved women, partners, and families experiencing perinatal loss [9,11,27].

Therefore, this umbrella review addressed two predefined objectives. The primary objective was to synthesize evidence from systematic reviews regarding the effectiveness of psychotherapeutic and psychosocial interventions for improving psychosocial outcomes following perinatal loss. The secondary exploratory objective was to examine how patient-centered care–related components—including therapeutic communication, patient engagement, therapeutic relationships, emotional validation, individualized support, and meaning-making processes—were described across the included interventions through secondary interpretive synthesis.

## 2. Materials and Methods

### 2.1. Design

An umbrella review of the published literature was conducted to synthesize evidence on psychotherapeutic interventions following perinatal loss and to explore intervention characteristics from a patient-centered care perspective. Umbrella reviews are a recognized form of evidence synthesis that integrate findings from multiple systematic reviews and meta-analyses on a common topic, providing a broad overview of the available evidence to inform healthcare practice and policy [32]. This methodological approach was selected because it enables a comprehensive evaluation of existing evidence and identifies consistencies, discrepancies, and gaps across published reviews.

This umbrella review was guided by one primary review question and one exploratory secondary question. The primary review question was: “What is the evidence from published systematic reviews regarding the effectiveness of psychotherapeutic and psychosocial interventions in improving psychosocial outcomes following perinatal loss?” Accordingly, the primary purpose of the review was to systematically identify, appraise, and synthesize evidence regarding psychotherapeutic interventions designed to alleviate psychological symptoms associated with perinatal loss, including grief, depression, anxiety, post-traumatic stress symptoms, psychological distress, coping, and related psychosocial outcomes.

The secondary exploratory review question was: “What patient-centered care–related characteristics are described within these interventions according to the included systematic reviews?” Accordingly, the review additionally examined how patient-centered care-related dimensions—including communication processes, therapeutic relationships, emotional validation, individualized support, patient engagement mechanisms, and meaning-making processes—were described across the included reviews, and how these recurring characteristics might help interpret psychosocial well-being following perinatal loss through a secondary interpretive synthesis.

The review was conducted in accordance with the methodological guidance for umbrella reviews developed by the Joanna Briggs Institute (JBI) [33,34]. All review procedures, including study identification, screening, eligibility assessment, quality appraisal, data extraction, and evidence synthesis, followed predefined methodological criteria to ensure transparency, consistency, and methodological rigor.

In addition to examining intervention effectiveness, the umbrella review methodology facilitated a secondary interpretive synthesis of intervention characteristics relevant to patient-centered care. Specifically, communication processes, therapeutic relationships, emotional validation, individualized support, patient engagement mechanisms, and meaning-making components reported within the included reviews were explored to provide a broader interpretive understanding of how communication-, support-, and engagement-related intervention characteristics were described in relation to psychosocial well-being following perinatal loss. Patient-centered care was used as an interpretive analytical framework during the synthesis and discussion of findings rather than as a primary search construct or eligibility criterion. This interpretive perspective did not modify the original review design, eligibility criteria, or study selection procedures.

The review protocol was prospectively registered in the International Prospective Register of Systematic Reviews (PROSPERO; Registration No.: CRD420261418238). The review question, eligibility criteria, search strategy, study selection procedures, quality appraisal process, data extraction methods, and synthesis approach were predefined in the registered protocol before data extraction and evidence synthesis.

### 2.2. Eligibility Criteria and Search Strategy

The umbrella review search strategy was developed in accordance with the methodological guidance of the Joanna Briggs Institute (JBI) and implemented in line with the procedures specified in the review protocol. Reporting of the search process followed the PRISMA 2020 statement [35] to enhance methodological transparency and reproducibility. Inclusion and exclusion criteria were established a priori using the PICOTSS framework (Population, Intervention, Comparator, Outcome, Time, Setting, and Study Characteristics) and are presented in Table 1. Because the review aimed primarily to synthesize evidence on psychotherapeutic interventions and psychosocial outcomes following perinatal loss, patient-centered care concepts were not treated as mandatory eligibility criteria but were examined subsequently through secondary interpretive synthesis. For consistency throughout this umbrella review, the term “bereaved parents” is used as an overarching descriptor encompassing women, partners, fathers, and couples affected by perinatal loss, unless the terminology of the original systematic reviews is reported explicitly. This terminology is adopted solely to improve consistency in describing the review population and does not alter the populations included in the original evidence syntheses.

The publication period was intentionally restricted to systematic reviews and meta-analyses published between 2019 and 2025 to synthesize the most recent and methodologically robust evidence. Over the past decade, psychotherapeutic approaches, digital interventions, and standards for evidence synthesis have evolved substantially. More recent reviews also incorporate a larger body of primary studies and generally reflect current methodological guidance for systematic reviews. Consequently, this review prioritized contemporary evidence that is more directly applicable to current clinical practice. Although earlier systematic reviews may have contributed historical insights, many of the primary studies included in earlier reviews are likely to have been incorporated into more recent evidence syntheses, thereby minimizing the risk of omitting influential evidence. Nevertheless, the possibility that some earlier reviews contained unique information not captured by more recent reviews cannot be entirely ruled out and is acknowledged as a limitation.

The literature search was conducted by C.K. Study selection was performed independently by C.K. and T.B. in two stages, including title/abstract screening and full-text eligibility assessment. Any disagreements regarding study inclusion were resolved through discussion until consensus was reached. Data extraction and methodological appraisal were undertaken by C.K. and T.B. using predefined criteria and standardized extraction fields to enhance consistency and transparency throughout the review process.

A systematic search was conducted in four electronic databases: the Cochrane Library, CINAHL, PubMed, and PsycINFO. The search covered eligible publications from database inception to 31 May 2026. Detailed search strategies for each database are provided in the Supplementary Online Material (Table S1: Cochrane Library, Table S2: CINAHL, Table S3: PubMed, and Table S4: PsycINFO). These databases were selected because they provide broad coverage of healthcare, psychology, mental health, and evidence synthesis literature.

The search strategy was designed to identify systematic reviews and meta-analyses that examined psychotherapeutic, psychosocial, or psychological support interventions following perinatal loss and reported psychosocial or psychological outcomes. Search terms combined controlled vocabulary (e.g., MeSH terms and database-specific subject headings) and free-text keywords covering three predefined conceptual domains: (1) perinatal loss (e.g., miscarriage, stillbirth, neonatal death, perinatal grief, fetal death), (2) psychotherapeutic and psychosocial interventions (e.g., psychotherapy, counseling, psychosocial support, bereavement support, cognitive behavioral therapy, mindfulness, crisis intervention), and (3) psychosocial outcomes (e.g., grief, bereavement, depression, anxiety, post-traumatic stress symptoms, psychological distress, coping, adjustment, and psychosocial well-being). To maximize retrieval sensitivity, outcome-related terminology was intentionally broad and incorporated multiple synonymous controlled vocabulary terms and free-text keywords across all databases. Consequently, the database-specific search strings included representative controlled vocabulary and free-text terms adapted to each database’s indexing system rather than identical terminology across all databases, and not every individual outcome term was expected to appear explicitly in every database-specific search string. Search terms were combined using Boolean operators (AND, OR) and adapted to the indexing structure and controlled vocabulary of each database. The complete database-specific search strategies are provided in the Supplementary Online Material (Tables S1–S4). An example of the search strategy applied in the Cochrane Library is presented in Table 2.

Patient-centered care–related concepts, including communication processes, therapeutic relationships, patient engagement, emotional validation, individualized support, and meaning-making, informed the secondary interpretive synthesis of the included reviews but were not applied as mandatory search filters or eligibility criteria. Accordingly, study selection was based primarily on psychotherapeutic interventions, perinatal loss, and psychosocial or psychological outcomes. At the same time, the patient-centered care perspective was applied during the later stages of evidence synthesis and interpretation.

### 2.3. Analytical Framework for Communication and Patient Engagement

In addition to the primary synthesis of intervention effectiveness, a secondary analytical framework was applied to explore communication- and engagement-related characteristics of the psychotherapeutic interventions reported within the included reviews. This framework was developed to support the patient-centered care perspective of the present umbrella review and focused on four dimensions: (a) mode of communication (individual, group-based, telephone-delivered, online, or blended interventions), (b) level of patient engagement (passive participation versus active involvement through exercises, self-reflection, or collaborative activities), (c) relational components (e.g., empathy, therapeutic alliance, trust, and continuity of support), and (d) narrative and meaning-making elements (e.g., expressive writing, storytelling, and reflective practices). The purpose of this supplementary synthesis was not to reassess intervention effectiveness, but to identify recurring communication- and engagement-related characteristics that could help contextualize psychosocial outcomes following perinatal loss from a patient-centered care perspective.

### 2.4. Coding Procedure

To support the secondary interpretive synthesis, all systematic reviews were independently examined by two reviewers (C.K. and T.B.). The coding process focused exclusively on the intervention characteristics described within the included systematic reviews rather than on the primary randomized controlled trials (RCTs) themselves. Specifically, the reviewers independently extracted and coded descriptions related to communication processes, patient engagement, therapeutic relationships, emotional validation, individualized support, accessibility and continuity of care, technology-assisted support, and meaning-making processes as reported by the review authors. This approach was intended to identify recurring patient-centered care–related characteristics across reviews without reinterpreting or reanalyzing the original primary studies. Following independent coding, the reviewers compared their coding decisions. Any discrepancies were resolved through discussion until full consensus was achieved. No formal inter-rater reliability statistic (e.g., Cohen’s κ) was calculated because the coding was intended to support an interpretive synthesis rather than quantitative content analysis. The agreed coding framework formed the basis for the secondary interpretive synthesis presented in the Results.

### 2.5. Coding Framework and Operational Definitions

To ensure methodological consistency and transparency during the secondary interpretive synthesis, a predefined coding framework was developed before coding. The framework was designed to identify patient-centered care–related characteristics explicitly described within the psychotherapeutic interventions reported in the included systematic reviews. Coding was performed exclusively at the review level and was based on the narrative descriptions, intervention characteristics, and synthesized findings presented by the review authors rather than on the original primary studies.

Eight patient-centered care dimensions were operationally defined a priori based on established patient-centered care principles and the objectives of the present umbrella review. Therapeutic communication was defined as explicit descriptions of structured therapeutic dialogue, counseling interactions, guided discussions, emotional expression, active listening, or other forms of purposeful clinician–patient communication. Patient engagement refers to the active involvement of participants in therapeutic activities, including structured writing exercises, self-reflection, collaborative tasks, mindfulness practices, homework assignments, and other components of participatory interventions. Individualized support was defined as the adaptation or tailoring of psychological care according to participants’ individual needs, preferences, or clinical circumstances. Emotional validation referred to intervention characteristics that explicitly acknowledged, accepted, normalized, or empathically responded to participants’ emotional experiences following perinatal loss. Therapeutic relationship encompassed descriptions of trust, rapport, empathy, continuity of care, therapeutic alliance, or supportive clinician–patient interactions. Meaning-making processes included intervention components facilitating reflection, narrative reconstruction, expressive writing, storytelling, or the reinterpretation of the loss experience. Accessibility and continuity of care referred to intervention characteristics intended to improve access to psychological support through flexible delivery methods or ongoing therapeutic contact. Finally, technology-assisted support was defined as the use of internet-based interventions, telehealth, telephone-delivered counseling, videoconferencing, or other digital communication platforms to support psychological care.

A patient-centered care dimension was assigned only when sufficient explicit narrative evidence describing the corresponding intervention characteristic was reported within a systematic review. Coding decisions were based on predefined operational definitions rather than subjective interpretation of intervention effectiveness. Where reporting was insufficient to determine whether a specific dimension was present, the characteristic was classified as unclear. Examples of coded intervention characteristics included therapist-guided counseling sessions, structured expressive writing exercises, mindfulness-based activities, internet-based cognitive behavioral therapy, telephone-delivered counseling, collaborative therapeutic exercises, and narrative reconstruction techniques, which were subsequently mapped to the corresponding patient-centered care dimensions presented in the Results. A patient-centered care dimension was coded as present (✓) only when explicit narrative descriptions corresponding to the predefined operational definition were identified within the review. General references to psychological support without sufficient descriptive detail were not considered sufficient to assign a positive code. Representative examples that support the operational coding framework are provided in Supplementary Table S6 to enhance the transparency and reproducibility of the secondary interpretive synthesis.

The coding approach employed in the present umbrella review should not be interpreted as a formal thematic synthesis of qualitative data. Rather, it represented a deductive, interpretive coding process based on predefined patient-centered care dimensions, applied to narrative descriptions of intervention characteristics reported in the included systematic reviews. This approach was intended to enhance transparency and consistency while supporting the review’s secondary exploratory objective.

### 2.6. Assessment of Overlap Between Ιncluded Reviews

To evaluate the degree of overlap among the primary studies included in the five eligible systematic reviews, a citation overlap matrix was constructed using the primary studies reported within the included reviews and the available article information. The Corrected Covered Area (CCA) was subsequently calculated in accordance with methodological recommendations for umbrella reviews [34] using the formula proposed by Pieper et al. [36]: CCA = (N − r)/(rc − r) where N represents the total number of study occurrences across all reviews, r the number of unique primary studies, and c the number of included systematic reviews. The degree of overlap was interpreted according to established thresholds: 0–5% = slight overlap; 6–10% = moderate overlap; 11–15% = high overlap; and >15% = very high overlap. Assessing overlap enabled evaluation of the potential duplication of primary evidence and informed interpretation of the synthesized findings. Because the overlap assessment was based on the primary studies reported within the included reviews and the available article information, it should be interpreted as an estimate of overlap across the available evidence syntheses rather than as an independent reappraisal of all original primary trial reports. The citation overlap matrix and CCA calculation are presented in Supplementary Table S7.

Where available, certainty-of-evidence assessments reported in the included systematic reviews (e.g., GRADE) were extracted and considered when interpreting the synthesized findings. No de novo certainty-of-evidence assessment was performed because the present study was an umbrella review synthesizing previously published systematic reviews rather than primary studies. Consequently, conclusions regarding intervention effectiveness were interpreted in light of both the methodological quality of the reviews (JBI appraisal) and any certainty-of-evidence evaluations reported by the original review authors.

## 3. Results

### 3.1. Study Selection

**Figure 1:**
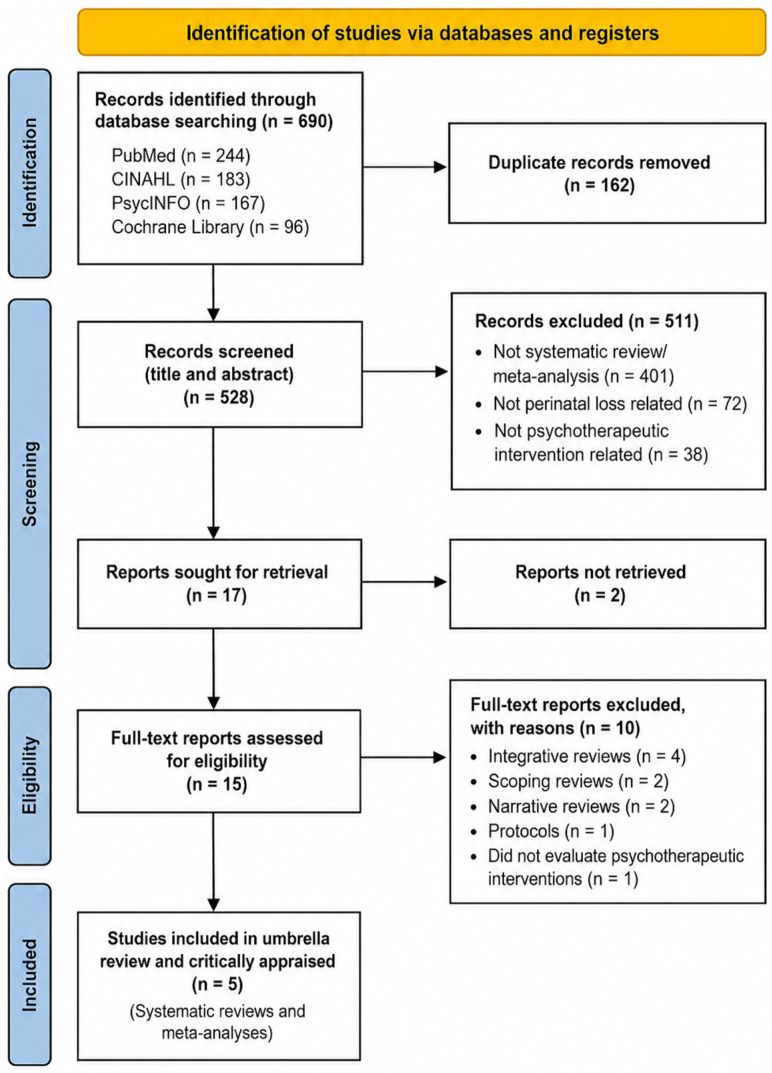
PRISMA flowchart outlining the selection process, critical appraisal, and data extraction.

The search strategy identified 690 records across the four electronic databases. After removing 162 duplicate records, 528 records were screened based on their titles and abstracts. Seventeen reports were sought for retrieval, and 15 full-text articles were assessed for eligibility. Following full-text assessment, 10 articles were excluded for failing to meet the predefined inclusion criteria. The reasons for exclusion are presented in Figure 1. Five systematic reviews and meta-analyses were subsequently included in the umbrella review and critically appraised using the Joanna Briggs Institute (JBI) Critical Appraisal Checklist for Systematic Reviews and Research Syntheses (Figure 1).

### 3.2. Characteristics of Included Reviews

Five reviews were included in the umbrella review: three systematic reviews and meta-analyses, one systematic review, and one network meta-analysis (Table 3). The reviews were published between 2021 and 2025 and focused on psychotherapeutic, psychosocial, and nonpharmacological interventions following perinatal loss. Three reviews were published in nursing and midwifery journals (International Journal of Nursing Studies, Journal of Midwifery & Women’s Health, and Worldviews on Evidence-Based Nursing). In contrast, two were published in broader mental health and bereavement journals (Depression and Anxiety and Omega—Journal of Death and Dying).

The included reviews searched between four and eight electronic databases, with PubMed/MEDLINE, CINAHL, PsycINFO, and the Cochrane Library being the most frequently used sources. Collectively, the reviews synthesized evidence from randomized controlled trials conducted across healthcare, community, and online settings in multiple countries. All five reviews evaluated psychotherapeutic or psychosocial interventions and reported beneficial effects on one or more psychosocial outcomes, including reduced symptoms of grief, depression, anxiety, and post-traumatic stress, as well as improvements in coping, adaptation, perceived social support, and broader psychosocial well-being following perinatal loss.

### 3.3. Methodological Quality of the Included Reviews

The methodological quality of the included reviews is summarized in Table S5. Methodological appraisal was conducted using the Joanna Briggs Institute (JBI) Critical Appraisal Checklist for Systematic Reviews and Research Syntheses. Overall, all five included reviews were judged to be of acceptable to high methodological quality, with most meeting 10 or 11 of the 11 appraisal criteria.

Minor methodological limitations were identified in two reviews. In the review by Shaohua and Shorey [37], the eligibility criteria were not explicitly structured according to a PICO framework, although the review objectives and inclusion criteria were clearly described. In the review by Dolan et al. [38], the assessment of publication bias was not clearly reported, resulting in some uncertainty for that appraisal item. Nevertheless, both reviews met most JBI checklist criteria and were considered sufficiently robust for inclusion in the present umbrella review.

Overall, the included reviews demonstrated clearly stated review questions, appropriate eligibility criteria, generally comprehensive search strategies, adequate sources of evidence, appropriate critical appraisal methods, and suitable approaches to evidence synthesis. Certainty-of-evidence assessments were reported inconsistently across the included reviews. Li et al. [22] explicitly evaluated certainty of evidence using the GRADE approach and reported moderate-to-high certainty for several psychosocial outcomes. Huang et al. [40] also incorporated certainty-of-evidence assessments based on the GRADE framework within their network meta-analysis. The remaining reviews primarily evaluated methodological quality and risk of bias but did not consistently report formal certainty-of-evidence assessments. Consequently, although the overall findings consistently supported the effectiveness of psychotherapeutic interventions, the certainty underlying these findings was interpreted with appropriate caution, particularly for outcomes derived from reviews that did not report formal certainty-of-evidence assessments. Although methodological appraisal supports confidence in the overall body of evidence, the findings should still be interpreted in light of differences across reviews in intervention content, populations, outcome measures, and reporting detail. Following appraisal, the findings of the included reviews were synthesized by psychosocial outcomes and by patient-centered care–related dimensions identified through secondary interpretive synthesis.

To further evaluate the methodological robustness of the umbrella review, overlap among the primary studies included in the five eligible systematic reviews was assessed using the Corrected Covered Area (CCA). The citation overlap matrix and corresponding CCA calculation are presented in Supplementary Table S7. The calculated CCA was 43.1%, indicating very high overlap according to established methodological thresholds. This finding suggests that several primary trials contributed to multiple systematic reviews and that some degree of duplication of primary evidence should be considered when interpreting the synthesized findings.

### 3.4. Effects of Psychotherapeutic Interventions on Psychosocial Outcomes

Across the five included reviews, psychotherapeutic and psychosocial interventions demonstrated generally positive effects on psychological and psychosocial outcomes following perinatal loss. The interventions evaluated included cognitive behavioral therapy (CBT), mindfulness-based interventions (MBIs), bereavement counseling, supportive counseling, psychosocial support programs, narrative-based interventions, problem-solving therapy, and digitally delivered psychological support interventions. Despite variations in intervention content, duration, delivery modality, and study populations, the overall direction of findings consistently indicated beneficial effects across multiple psychosocial outcomes. A summary of the psychosocial outcomes reported across the included reviews is presented in Table 4.

Improvements in grief-related outcomes were reported across all included reviews. Shaohua and Shorey [37] demonstrated significant reductions in grief symptoms following psychosocial interventions, while Dolan et al. [38] reported beneficial effects of both CBT and mindfulness-based approaches on complicated perinatal grief—similarly, Li et al. [22] found significant improvements in grief among parents receiving nonpharmacological interventions. Karaahmet and Bilgiç [39] reported enhanced grief adaptation among women following stillbirth, whereas Huang et al. [40] identified bereavement support interventions as the most effective approach for grief reduction based on network meta-analysis findings.

Depression and anxiety were among the most frequently evaluated outcomes. All five reviews reported favorable effects on depressive symptoms, with several studies demonstrating statistically significant reductions following psychotherapeutic interventions. Similar improvements were observed for anxiety, particularly among participants receiving cognitive-based interventions, supportive counseling, and problem-solving therapy—the network meta-analysis conducted by Huang et al. [40] indicated that problem-solving therapy achieved the highest ranking for reducing both depression and anxiety among women experiencing perinatal loss.

Reduced post-traumatic stress symptoms and improvements in broader indicators of psychological distress were also reported. Dolan et al. [38] reported reductions in post-traumatic stress symptoms following both CBT and mindfulness-based interventions, while Li et al. [22] identified significant improvements in post-traumatic stress outcomes across multiple nonpharmacological interventions. Karaahmet and Bilgiç [39] further demonstrated reductions in stress levels among women receiving psychotherapy after stillbirth. Collectively, these findings suggest that psychotherapeutic interventions may facilitate emotional recovery and adaptation following traumatic reproductive experiences.

Several reviews additionally highlighted improvements in perceived social support, coping, emotional adaptation, and overall psychosocial well-being. Li et al. [22] reported significant gains in perceived social support, whereas multiple interventions incorporated therapeutic and supportive elements to strengthen emotional processing, resilience, and adjustment to loss. Face-to-face, individualized, and cognitive-based interventions appeared particularly beneficial across several outcomes. Furthermore, both traditional and technology-assisted interventions appear to demonstrate positive effects, suggesting that psychological support can be delivered through a range of modalities.

Overall, the synthesized evidence indicates generally consistent beneficial effects across reviews, although certainty of evidence varied according to the methodological assessments reported in the original reviews. Although intervention characteristics varied considerably across reviews, the consistency of these beneficial effects supports integrating structured psychological support into routine perinatal bereavement care.

### 3.5. Patient-Centered Care Dimensions Within Psychotherapeutic Interventions

Patient-centered care emphasizes delivering healthcare that respects individuals’ preferences, values, experiences, and psychosocial needs while promoting active participation in care processes. Across the included reviews, several communication-, support-, and engagement-related characteristics consistent with a patient-centered care perspective were identified within psychotherapeutic and psychosocial interventions following perinatal loss. Although these dimensions were not typically evaluated as primary outcomes, they emerged as recurring interpretive features of interventions described as beneficial across the included reviews.

A prominent finding across the reviews was the central role of therapeutic communication. Counseling, psychotherapy, and bereavement support interventions provided structured opportunities for participants to express emotions, discuss loss-related experiences, and explore personal meanings associated with bereavement. Interventions based on cognitive-behavioral therapy, narrative approaches, and supportive counseling frequently relied on open communication, emotional disclosure, and guided reflection as recurring therapeutic features.

A second recurring dimension was active patient engagement. Several interventions encouraged participants to take an active role in the therapeutic process through structured writing exercises, self-reflection activities, mindfulness practices, goal-oriented tasks, and collaborative therapeutic work. Such approaches were described as incorporating active patient involvement and were frequently reported in interventions associated with beneficial psychosocial outcomes. Individualized support and emotional validation also emerged as important characteristics of effective interventions. Many programs were tailored to participants’ emotional needs, grief experiences, and psychological symptoms. Counseling-based interventions frequently emphasize empathy, acceptance, and validation of emotional responses, thereby fostering a supportive therapeutic environment. These relational components were frequently described across interventions identified as beneficial. Finally, several reviews highlighted the contribution of accessible and flexible models of care, including internet-based cognitive behavioral therapy, telephone counseling, and other technology-assisted interventions. These approaches were described as improving access to psychological support and facilitated continuity of care for individuals who might otherwise face geographical, practical, or emotional barriers to accessing services.

Overall, the included reviews consistently described communication-, support-, and engagement-related characteristics within interventions associated with beneficial psychosocial outcomes. These recurring characteristics formed the basis of the secondary interpretive synthesis and may provide a useful patient-centered care perspective for interpreting psychosocial adaptation following perinatal loss. However, they were not directly evaluated as mechanisms of intervention effectiveness. A summary of these dimensions is presented in Table 5. These findings provide the basis for the subsequent discussion regarding the potential role of a patient-centered care perspective in understanding psychosocial well-being following perinatal loss. These dimensions should be interpreted as recurring intervention characteristics identified through secondary interpretive synthesis rather than directly measured patient-centered care outcomes.

### 3.6. Communication and Patient Engagement Characteristics of Interventions

Across the included reviews, several communication- and engagement-related characteristics were described within psychotherapeutic interventions following perinatal loss. Cognitive behavioral therapy (CBT)-based interventions typically involved structured communication processes and often required active patient participation through self-monitoring, written exercises, behavioral tasks, and reflective activities. Mindfulness-based interventions commonly encouraged experiential engagement and self-awareness through guided reflection and emotional regulation practices. Counseling-based interventions were generally characterized by relational communication processes, including emotional expression, empathy, active listening, and therapeutic support. Collectively, these descriptions indicate that trust-building and supportive therapeutic relationships were recurrent intervention characteristics reported across the included reviews.

Several interventions also incorporated digital communication modalities, including telephone counseling and internet-based therapeutic approaches. These delivery methods appeared to facilitate accessibility, continuity of support, and ongoing engagement, particularly for individuals experiencing barriers to face-to-face care. Narrative and expressive components, such as writing activities, storytelling, and opportunities for emotional disclosure, were also described across several interventions. From a patient-centered care perspective, these elements may help interpret how certain interventions support meaning-making processes and psychological adaptation following perinatal loss.

**Figure 2:**
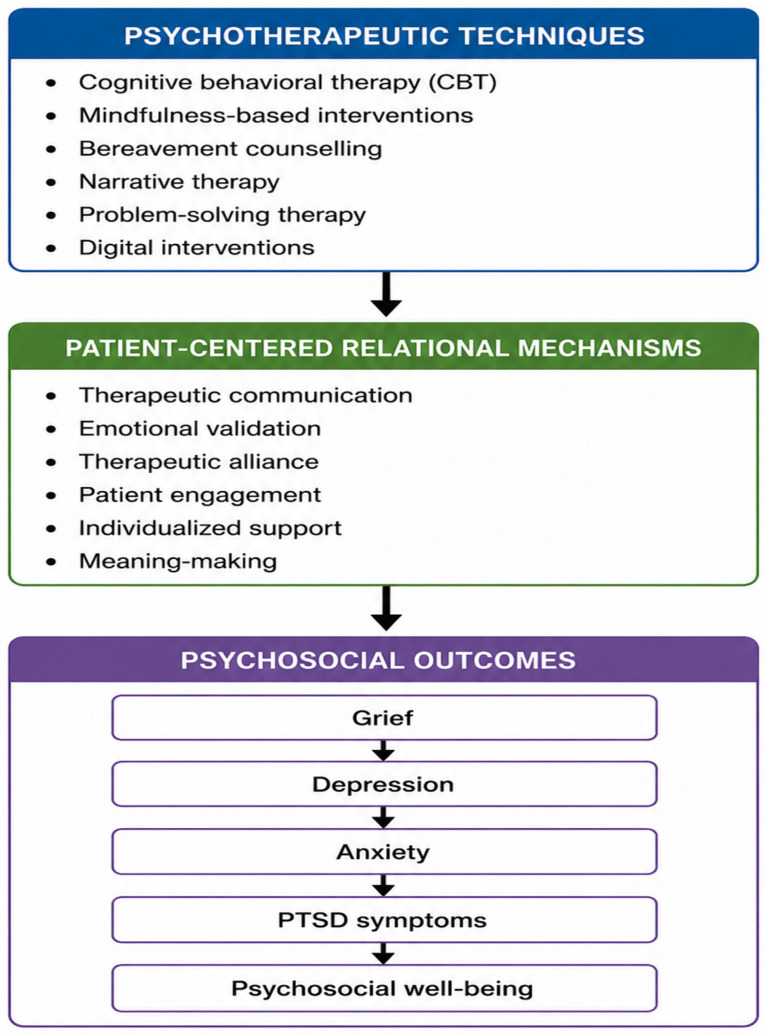
Conceptual framework illustrating communication processes and patient engagement characteristics across psychotherapeutic interventions following perinatal loss. The figure summarizes how major intervention categories (cognitive behavioral therapy, mindfulness-based interventions, counseling, narrative interventions, and digital psychological support) incorporate therapeutic communication, patient engagement, therapeutic relationships, emotional validation, and meaning-making processes that may contribute to improved psychosocial outcomes within a patient-centered care perspective.

To facilitate the interpretation of the secondary synthesis, Figure 2 summarizes this interpretive framework by illustrating how the psychotherapeutic interventions synthesized in this umbrella review shared several recurring patient-centered characteristics despite differences in therapeutic orientation. Across intervention types, structured therapeutic communication, opportunities for active patient engagement, supportive therapeutic relationships, emotional validation, and meaning-making processes consistently emerged as common descriptive characteristics identified across interventions associated with improvements in grief, depression, anxiety, post-traumatic stress symptoms, and broader psychosocial well-being. The conceptual framework complements the quantitative synthesis by illustrating how patient-centered care-related characteristics were described across interventions following perinatal loss.

## 4. Discussion

This umbrella review synthesizes evidence from five systematic reviews and meta-analyses examining psychotherapeutic and psychosocial interventions to improve psychological outcomes following perinatal loss. By integrating findings across reviews that differ in scope, intervention characteristics, populations, and methodological approaches, the present review provides a broader understanding of how psychological support may facilitate adaptation following perinatal loss. Whereas individual reviews primarily focused on intervention effectiveness, the current synthesis additionally examined communication-, support-, and engagement-related characteristics described across these interventions from a patient-centered care perspective, including therapeutic communication, emotional validation, individualized support, patient engagement, and meaning-making processes. Importantly, a distinction should be made between the psychotherapeutic techniques employed (e.g., cognitive behavioral therapy, mindfulness-based interventions, bereavement counseling, and narrative interventions) and the patient-centered relational characteristics identified across these interventions (e.g., therapeutic communication, emotional validation, therapeutic alliance, patient engagement, individualized support, and meaning-making). The former represent intervention modalities, whereas the latter emerged through the secondary interpretive synthesis as recurring relational characteristics described within interventions rather than empirically evaluated mechanisms of therapeutic effectiveness. In doing so, the review offers a broader interpretive perspective on how such characteristics may relate to psychosocial well-being following perinatal loss. Importantly, this patient-centered care perspective should be viewed as an interpretive conceptual framework applied during evidence synthesis rather than as an empirical evaluation of patient-centered care processes within the included reviews. As illustrated in Figure 2, the psychotherapeutic interventions synthesized in this umbrella review shared several recurring patient-centered characteristics despite differences in therapeutic orientation. Across intervention types, structured therapeutic communication, opportunities for active patient engagement, supportive therapeutic relationships, emotional validation, and meaning-making processes consistently emerged as common descriptive characteristics identified across interventions associated with improvements in grief, depression, anxiety, post-traumatic stress symptoms, and broader psychosocial well-being. The conceptual framework therefore complements the quantitative synthesis by illustrating how patient-centered care-related characteristics were described across interventions following perinatal loss.

A central finding emerging from this synthesis is the consistent effectiveness of psychotherapeutic and psychosocial interventions in reducing grief, depression, anxiety, post-traumatic stress symptoms, and broader indicators of psychological distress. Across all included reviews, interventions such as cognitive behavioral therapy, mindfulness-based approaches, bereavement counseling, supportive counseling, and problem-solving therapy demonstrated beneficial effects on at least one major psychosocial outcome. Similar conclusions were reported by Shaohua and Shorey [37], who found that psychosocial interventions were associated with significant improvements in grief, depression, and anxiety among bereaved parents. Likewise, Dolan and colleagues [38] reported positive effects of both cognitive behavioral therapy and mindfulness-based interventions on complicated perinatal grief, while Li and colleagues [22] demonstrated that nonpharmacological interventions were associated with improvements in grief, post-traumatic stress symptoms, depression, anxiety, and perceived social support. More recent evidence from Karaahmet and Bilgiç [39] and Huang and colleagues [40] further supports the effectiveness of psychotherapy and psychosocial support interventions in facilitating emotional adaptation after perinatal loss.

The consistency of these findings is particularly important given the substantial psychological burden associated with perinatal bereavement. Previous literature has demonstrated that bereaved parents and their families are at increased risk of prolonged grief, depression, anxiety, and post-traumatic stress reactions that may persist for months or even years following the loss. Kersting and Wagner [3] highlighted the complex and multifaceted nature of perinatal grief, whereas Lok and Neugebauer described the elevated psychological morbidity observed after miscarriage. More recently, Kukulskienė and Žemaitienė [6], Lamon and colleagues [7], and Donegan and colleagues [9] reported persistent emotional distress among bereaved parents (including women, fathers, partners, and couples) following perinatal loss. The present umbrella review therefore reinforces the growing consensus that structured psychological support may represent an important component of comprehensive perinatal bereavement care rather than an optional adjunct service.

Beyond assessing intervention effectiveness, this review makes an important contribution by examining patient-centered care–related dimensions described across psychotherapeutic interventions following perinatal loss. Although none of the included reviews explicitly evaluated patient-centered care as a primary outcome, several recurring characteristics were evident across interventions described as beneficial. Counseling-based approaches, narrative interventions, structured writing exercises, mindfulness activities, and supportive psychotherapy were consistently described as incorporating communication processes that enabled individuals to express emotions, explore loss-related experiences, and reconstruct personal meaning following bereavement. These observations are consistent with the conceptual framework proposed by Epstein and Street [29], who describe patient-centered care as a process through which healthcare professionals acknowledge individual experiences, preferences, and emotional needs while promoting meaningful engagement in care.

Recent systematic reviews have also highlighted the importance of using validated instruments to evaluate patient-centered care and related communication processes in clinical practice [41,42]. Evidence syntheses evaluating patient-centered care measurement instruments have demonstrated that although numerous tools are available, important differences exist regarding their psychometric quality, reliability, content validity, and conceptual coverage [41,43]. Furthermore, methodological guidance based on the COSMIN framework emphasizes the importance of selecting measurement instruments with demonstrated validity and reliability when evaluating complex multidimensional constructs such as patient-centered care [42]. These findings reinforce that patient-centered care should ideally be assessed using standardized and psychometrically robust instruments rather than inferred solely from intervention descriptions. Consequently, the present interpretive synthesis should be viewed as a conceptual framework that may inform future studies incorporating validated patient-centered care measures alongside psychosocial outcomes.

Therapeutic communication was one of the most consistently described intervention characteristics across the included interventions. Whether delivered face-to-face, by telephone, or through digital platforms, interventions described as beneficial commonly incorporated opportunities for emotional disclosure, empathetic listening, and collaborative exploration of grief experiences. Street and colleagues [27] have emphasized that communication is a central determinant of patient outcomes, as it influences trust, emotional adjustment, treatment engagement, and shared understanding. Similarly, the therapeutic alliance has repeatedly been identified as one of the strongest predictors of successful psychotherapy outcomes across diverse mental health conditions. The meta-analytic findings of Flückiger and colleagues [30] support this interpretation by demonstrating a robust association between therapeutic alliance and treatment effectiveness. In the context of the present umbrella review, these observations do not demonstrate causal mechanisms but suggest that communication-related characteristics were consistently described across interventions associated with beneficial psychosocial outcomes.

Another notable finding concerns the role of patient engagement and active participation in the recovery process. Several interventions identified within the included reviews incorporated structured writing exercises, self-reflection activities, mindfulness practices, and collaborative therapeutic tasks that required participants to take an active role in treatment. This observation aligns closely with contemporary patient-centered care models, which emphasize partnership, empowerment, and shared decision-making. Barello and Graffigna [28] have argued that patient engagement represents a critical pathway through which healthcare interventions improve outcomes, while Barry and Edgman-Levitan [31] identified shared participation as a cornerstone of patient-centered care. The present interpretive synthesis indicates that interventions promoting active involvement were recurrently described across interventions associated with emotional adaptation following perinatal bereavement.

The psychosocial mechanisms identified through the present interpretive synthesis are also consistent with broader evidence derived from caregiving and bereavement research beyond the context of perinatal loss. Contemporary patient-centered care frameworks emphasize that emotional validation, effective communication, individualized support, therapeutic relationships, patient engagement, and shared decision-making are fundamental determinants of psychological adjustment across diverse healthcare settings [25,27,28,29,30,31]. Similarly, systematic reviews conducted in caregiver populations have consistently reported associations between psychosocial adaptation and caregiver burden, perceived stress, quality of life, resilience, coping resources, and social support [44,45,46,47]. Although these studies involve different clinical populations, they support the broader theoretical interpretation that the communication-, engagement-, and support-related characteristics identified in the present umbrella review reflect common psychosocial mechanisms that may extend beyond the specific context of perinatal bereavement.

The increasing use of digitally delivered interventions represents another important development identified in this review. Internet-based cognitive behavioral therapy, online counseling programs, telephone-delivered interventions, and technology-assisted psychological support demonstrated positive effects across several psychosocial outcomes. Such approaches may address practical barriers that frequently limit access to bereavement services, including geographical distance, time constraints, stigma, and limited availability of specialist providers. These findings are consistent with broader healthcare trends emphasizing accessibility, flexibility, and continuity of care [25,31]. Nevertheless, while digital interventions appear promising, further research is required to determine which patients benefit most from remote delivery models and whether specific therapeutic components are more effectively delivered through face-to-face or digital formats. From a clinical perspective, the findings support the routine integration of psychological assessment and referral pathways within maternity and bereavement services following perinatal loss.

Recent evidence further suggests that digitally delivered bereavement support should be considered as a complement rather than a replacement for human-centered psychological care. A recent systematic review of digital grief technologies by Soh et al. [48] highlighted several potential benefits of technology-assisted bereavement support, including improved accessibility, flexibility, continuity of care, and opportunities for ongoing emotional support. At the same time, the authors emphasized important challenges related to emotional distance, privacy and confidentiality, digital inequities, overreliance on technology, and the limitations of replacing therapeutic human relationships. These observations are consistent with the present umbrella review, in which technology-assisted interventions appeared to enhance access and continuity of care but should be interpreted within a broader patient-centered framework that continues to prioritize therapeutic communication, empathy, and interpersonal relationships.

An additional consideration concerns the acceptability and timing of psychotherapeutic interventions following perinatal loss. Although the reviews included generally reported beneficial psychosocial outcomes, psychological support may not be equally acceptable or appropriate for all bereaved parents at every stage of the grieving process. Some individuals may not feel emotionally ready to engage in structured psychological interventions immediately after the loss, whereas others may prefer informal support, family-centered care, peer support, or a period of adjustment before participating in psychotherapy. Furthermore, emotionally intensive interventions may temporarily increase distress as grief-related experiences are revisited, and treatment discontinuation or reduced engagement may occur when interventions are perceived as poorly timed or insufficiently tailored to individual needs. These observations reinforce the importance of adopting a patient-centered approach in which intervention timing, mode of delivery, therapeutic intensity, and patient preferences are considered collaboratively rather than assuming that one intervention strategy is universally appropriate for all bereaved parents. The considerable heterogeneity across the included reviews should also be considered when interpreting the findings. Intervention duration, intensity, delivery modality (face-to-face, telephone, or digital), participant characteristics (e.g., women, fathers, couples), type of perinatal loss (miscarriage, stillbirth, or neonatal death), and the timing of intervention initiation following the loss varied substantially across studies. These methodological and clinical sources of heterogeneity may partly explain the variability in reported intervention effects and limit direct comparisons between therapeutic approaches. Consequently, although the overall direction of evidence supports the effectiveness of psychotherapeutic interventions, caution is warranted when interpreting comparative effectiveness across different intervention models.

Finally, this umbrella review highlights important gaps within the current evidence base. Although psychotherapeutic interventions consistently demonstrated positive outcomes, the included reviews varied considerably regarding intervention content, duration, intensity, delivery format, and outcome measurement. Furthermore, most studies evaluated symptom reduction rather than patient-centered outcomes such as communication quality, patient experience, engagement, empowerment, therapeutic alliance, or shared decision-making. Consequently, while the present synthesis suggests that patient-centered care–related dimensions may help interpret psychological recovery, direct empirical evidence remains limited. Future research should therefore move beyond evaluating intervention effectiveness alone and should also examine intervention acceptability, patient preferences, treatment adherence, reasons for non-participation or dropout, and the optimal timing for initiating psychological support following perinatal loss.

### 4.1. Implications

The findings of this umbrella review have several implications for clinical practice, perinatal bereavement care, healthcare systems, and future research. In clinical practice, psychotherapeutic and psychosocial interventions appear to represent important components of care following perinatal loss rather than optional supportive services alone. The beneficial effects reported across the included reviews for grief, depression, anxiety, post-traumatic stress symptoms, and psychosocial well-being suggest that structured psychological support may play a meaningful role in facilitating emotional recovery among bereaved parents. Interventions that promote active participation, emotional expression, and individualized support may be particularly valuable, as they address both the psychological consequences of loss and the highly personal experiences associated with bereavement. Clinical decision-making should also consider patient readiness, preferences, and the acceptability of different intervention modalities when planning psychological support following perinatal loss.

From a patient-centered care perspective, healthcare professionals involved in perinatal bereavement care may prioritize communication processes that facilitate empathy, emotional validation, shared understanding, and therapeutic engagement. According to patient-centered care frameworks, the therapeutic alliance, encourage emotional disclosure, and support meaning-making processes relevant to psychological adaptation following loss. The findings of this review further indicate that interventions may be more responsive when tailored to the individual needs, preferences, and circumstances of bereaved parents rather than relying solely on standardized approaches. Incorporating patient-centered principles into bereavement care may improve patient experiences and support psychosocial outcomes, while promoting more compassionate and responsive healthcare delivery.

At the healthcare system level, the findings may support integrating psychological support services into routine maternity, obstetric, neonatal, and community healthcare pathways. Many individuals experiencing perinatal loss encounter barriers to accessing specialized psychological care, including geographical limitations, service availability, financial constraints, and stigma surrounding mental health support. The positive findings reported for internet-based cognitive behavioral therapy, telephone counseling, and other technology-assisted interventions suggest that digital models of care may represent viable approaches to expanding access to psychological support. Consistent with patient-centered care frameworks, healthcare organizations should seek to develop flexible service models that improve accessibility, continuity of care, and responsiveness to individual patient needs. Nevertheless, digital approaches should complement rather than replace face-to-face therapeutic relationships, particularly in the emotionally complex context of perinatal bereavement, where empathy, trust, and individualized communication remain fundamental components of patient-centered care.

Several implications for intervention development also emerge from the present synthesis. Future psychological support programs should move beyond symptom reduction alone and more explicitly consider communication-, support-, and engagement-related characteristics, including therapeutic communication, patient engagement, emotional validation, individualized support, and collaborative goal setting. Although these elements frequently appeared in interventions described as beneficial, they were rarely evaluated directly as components of the interventions or as outcome measures. Greater attention to these relational and communicative aspects may facilitate the development of more comprehensive models of perinatal bereavement care.

Finally, several research priorities emerge from the current evidence base. Future studies should examine how psychotherapeutic interventions achieve their beneficial effects, particularly the role of communication quality, therapeutic alliance, patient engagement, and meaning-making processes. There is also a need for greater standardization of outcome measures to facilitate comparisons across studies and intervention types. Additional high-quality randomized controlled trials are needed to evaluate emerging interventions, including digitally delivered therapies and integrated models of psychological support. Furthermore, longitudinal research is needed to determine the sustainability of intervention effects over time and to identify which approaches are most effective for specific populations experiencing different forms of perinatal loss. Such evidence would support the development of more targeted, accessible, and patient-centered models of psychological care for bereaved parents and families.

### 4.2. Strengths and Limitations

To our knowledge, this is the first umbrella review to systematically synthesize evidence on psychotherapeutic interventions following perinatal loss and to examine these interventions through a patient-centered care perspective. A comprehensive search strategy was conducted across four major electronic databases (Cochrane Library, CINAHL, PubMed, and PsycINFO), and the methodological quality of the included reviews was appraised using the Joanna Briggs Institute (JBI) Critical Appraisal Checklist for Systematic Reviews and Research Syntheses in accordance with contemporary guidance for umbrella reviews [32,34]. Furthermore, the review followed the PRISMA 2020 reporting recommendations to enhance methodological transparency and reproducibility [35]. While previous systematic reviews and meta-analyses primarily examined the effectiveness of psychotherapeutic interventions on grief, depression, anxiety, post-traumatic stress symptoms, and related psychosocial outcomes [22,37,38,39,40], the present umbrella review additionally explored communication-, support-, and engagement-related characteristics from a patient-centered care perspective, thereby providing a broader interpretive understanding of how such characteristics may relate to psychological adaptation following perinatal loss.

Despite these strengths, several limitations should be acknowledged. First, although extensive efforts were undertaken to identify all relevant systematic reviews and meta-analyses, the umbrella review design relies exclusively on previously published secondary research. Consequently, recently published primary studies, emerging interventions, and newly developed models of perinatal bereavement support may not have been captured unless they were already included in an eligible systematic review. This structural limitation is inherent to umbrella reviews and may result in a temporal lag between the publication of primary evidence and its incorporation into higher-level evidence syntheses [32]. Moreover, because umbrella reviews synthesize published systematic reviews rather than primary studies directly, any publication bias present within the included reviews may have been inherited by the present synthesis and could have influenced the overall conclusions.

Second, the findings of this review were constrained by the methodological and reporting characteristics of the included reviews. Considerable heterogeneity was observed regarding intervention content, duration, intensity, delivery format, participant characteristics, and outcome measures. The included reviews evaluated a wide range of interventions, including cognitive-behavioral therapy, mindfulness-based interventions, bereavement counseling, supportive psychotherapy, problem-solving therapy, and digitally delivered support programs [22,37,38,39,40]. Such variability limited the possibility of direct comparisons across interventions and reduced the feasibility of drawing definitive conclusions regarding the superiority of any single therapeutic approach. Furthermore, psychosocial outcomes were assessed using a variety of measurement instruments across the included reviews, limiting direct comparisons between intervention effects and reducing methodological consistency across the synthesized evidence. In addition, the primary studies included within the reviewed systematic reviews were conducted across diverse countries and healthcare systems. Cultural differences in beliefs surrounding pregnancy loss, bereavement practices, family involvement, and help-seeking behaviors may have influenced both the acceptability and effectiveness of psychotherapeutic interventions, thereby limiting the generalizability of the findings across different cultural contexts.

Third, although the present review sought to examine patient-centered care dimensions within psychotherapeutic interventions, these constructs were rarely measured directly within the included reviews. Communication quality, therapeutic alliance, emotional validation, patient engagement, and meaning-making processes were generally inferred from intervention descriptions rather than evaluated as primary outcomes. Consequently, the patient-centered care framework employed in this review should be interpreted as an analytical and interpretive lens rather than as a directly measured intervention outcome. While this approach is supported by contemporary conceptualizations of patient-centered care [25,29], direct empirical evidence regarding the contribution of specific patient-centered mechanisms remains limited. Although predefined operational definitions, independent coding by two reviewers, and consensus procedures were applied to improve consistency, the secondary interpretive synthesis inevitably involved a degree of subjective judgement. Accordingly, alternative conceptual frameworks or coding approaches might have yielded somewhat different interpretations of the intervention characteristics.

Fourth, several methodological considerations regarding the search strategy warrant attention. The review was restricted to publications available in English and to systematic reviews and meta-analyses published between 2019 and 2025. Although the latter restriction was intended to prioritize the most recent and methodologically robust evidence syntheses, it may have resulted in the exclusion of earlier systematic reviews that contained unique evidence not subsequently incorporated into more recent reviews. In addition, restricting the review to English-language publications may have resulted in the omission of relevant evidence published in other languages or presented in alternative forms of evidence synthesis. Furthermore, although efforts were made to use broad search terms related to perinatal loss, psychotherapy, psychosocial support, and psychological outcomes, variations in terminology across disciplines may have resulted in the omission of some relevant studies. Finally, overlap among primary studies included within multiple systematic reviews represents a potential source of duplication bias. To evaluate this issue, overlap was formally assessed using the Corrected Covered Area (CCA), which indicated very high overlap (43.1%) among the included reviews (Supplementary Table S7). This finding suggests that several influential primary studies contributed to more than one systematic review and should therefore be considered when interpreting the synthesized evidence. Nevertheless, because the present umbrella review synthesized findings at the review level rather than reanalyzing individual primary studies, the impact of duplicate evidence on the overall conclusions is likely to be limited, although the findings should still be interpreted with appropriate caution. In addition, the relatively small number of eligible systematic reviews identified highlights the need for further high-quality evidence syntheses and primary intervention studies in the field of perinatal bereavement care. An additional limitation should be acknowledged. Communication processes, patient engagement mechanisms, and therapeutic alliance variables were not directly measured in the primary studies included within the reviewed systematic reviews. Consequently, the communication-focused synthesis presented in this umbrella review was based on the interpretation of intervention characteristics reported by the original authors rather than on direct empirical measurement. Therefore, these findings should be interpreted with appropriate caution and regarded as exploratory. Finally, the included reviews provided limited information regarding intervention acceptability, treatment adherence, participant dropout, and patient preferences. Consequently, the present umbrella review was unable to synthesize these aspects systematically, highlighting an important area for future research. Furthermore, certainty-of-evidence assessments were not consistently reported across all included systematic reviews. Although certainty-of-evidence assessments reported by the original reviews (e.g., GRADE) were considered during interpretation, the absence of standardized certainty-of-evidence assessments across all included reviews limits the strength with which conclusions regarding intervention effectiveness can be drawn.

Despite these limitations, the methodological quality of the reviews included was generally high, and the consistency of findings across reviews strengthens confidence in the overall conclusions. The present umbrella review therefore provides a robust synthesis of current evidence on psychotherapeutic interventions. It offers novel insights into the potential role of dimensions of patient-centered care in promoting psychosocial well-being following perinatal loss.

## 5. Conclusions

This umbrella review synthesizes evidence from five systematic reviews and meta-analyses that examine psychotherapeutic and psychosocial interventions to support bereaved parents following perinatal loss. The findings indicate that a range of interventions, including cognitive behavioral therapy, mindfulness-based approaches, bereavement counseling, supportive psychotherapy, problem-solving therapy, and digitally delivered psychological support, are generally associated with improvements in grief, depression, anxiety, post-traumatic stress symptoms, and broader indicators of psychosocial well-being. Collectively, the evidence supports the inclusion of structured psychological support within perinatal bereavement care.

Beyond intervention effectiveness, this review highlights the possible relevance of a patient-centered care perspective for interpreting psychological adaptation following perinatal loss. Therapeutic communication, emotional validation, patient engagement, individualized support, therapeutic relationships, and meaning-making processes appeared as recurring characteristics described across interventions identified as beneficial. However, these dimensions were rarely evaluated directly as intervention outcomes or as empirically tested mechanisms of effect. Accordingly, they should be interpreted as patient-centered care–related characteristics identified through secondary interpretive synthesis rather than as confirmed mechanisms of intervention effectiveness. Accordingly, these findings should be interpreted as descriptive characteristics identified across interventions rather than empirical evidence of causal pathways linking patient-centered care processes with psychosocial outcomes.

For clinical practice, the findings underscore the importance of providing accessible, individualized, and compassionate psychological support that extends beyond symptom management alone. Healthcare professionals may adopt patient-centered approaches that encourage active participation, acknowledge the unique experiences of bereaved parents, and foster supportive therapeutic relationships throughout the grieving process. The growing evidence supporting digitally delivered interventions further highlights opportunities to improve access, continuity of care, and service availability for individuals who may encounter barriers to traditional forms of support.

At the same time, important evidence gaps remain. Future research should move beyond evaluating intervention effectiveness alone and examine how communication processes, therapeutic alliance, patient engagement, and other patient-centered care–related dimensions influence psychosocial outcomes following perinatal loss. Future studies should also investigate whether therapeutic alliance is associated with treatment effectiveness, directly assess communication quality using validated instruments, and incorporate validated patient-centered care measures to enable more rigorous evaluation of patient-centered care processes. Greater standardization of validated psychosocial outcome measures, high-quality randomized controlled trials, and longitudinal studies examining the sustainability of intervention effects are also needed. Strengthening this evidence base will support the development of more effective, accessible, and patient-centered models of perinatal bereavement care to improve psychological outcomes and psychosocial well-being among bereaved parents and their families.

## Figures and Tables

**Table 1:** Selection criteria for inclusion or exclusion of reviews.

	Inclusion Criteria	Exclusion Criteria
Population	Bereaved parents (women, partners, or couples) aged ≥18 years who experienced perinatal loss.	Participants younger than 18 years
Intervention	Psychotherapeutic interventions, psychological counseling, psychotherapy, psychosocial support, bereavement counseling, grief-focused interventions, psychoeducation with a psychotherapeutic component, cognitive-behavioral interventions, mindfulness-based interventions, digitally delivered psychological support programs, and other structured psychological or psychosocial support interventions.	Pharmacological interventions, educational interventions without a psychotherapeutic component, and non-psychological supportive interventions
Comparator	Different psychotherapeutic approaches, usual care, no intervention, wait-list controls, or alternative supportive interventions	
Outcome Measures	Anxiety, depression, grief, complicated grief, post-traumatic stress symptoms, psychological distress, psychosocial well-being, emotional adaptation, coping, perceived social support, and other psychosocial or psychological outcomes following perinatal loss.	Studies that do not report psychosocial or psychological outcomes.
Time	Published between 2019 and 2025	Published before 2019
Setting	All healthcare and community settings	Not restricted
Study Characteristics	Systematic reviews and meta-analyses (secondary research)	Narrative reviews, scoping reviews, protocols, editorials, commentaries, dissertations, conference abstracts, and grey literature
Language of Publication	Abstract in English; full text in English or Greek	Other languages

**Table 2:** Illustrative search strategy (Cochrane Library; searches conducted from database inception to 31 May 2026).

Search ID	Search Term	Results
#1	MeSH descriptor: [Psychosocial Intervention] explode all trees	241
#2	MeSH descriptor: [Internet-Based Intervention] explode all trees	573
#3	MeSH descriptor: [Crisis Intervention] explode all trees	319
#4	MeSH descriptor: [Psychotherapy] explode all trees	33,845
#5	MeSH descriptor: [Anxiety] explode all trees	13,389
#6	MeSH descriptor: [Anxiety Disorders] explode all trees	9296
#7	MeSH descriptor: [Depression] explode all trees	18,834
#8	MeSH descriptor: [Depressive Disorder] explode all trees	15,381
#9	MeSH descriptor: [Stress Disorders, Traumatic, Acute] explode all trees	58
#10	MeSH descriptor: [Abortion, Spontaneous] explode all trees	1228
#11	MeSH descriptor: [Fetal Death] explode all trees	549
#12	MeSH descriptor: [Systematic Review] explode all trees	426
#13	psychotherap* OR counsel* OR psychosocial intervention* OR crisis intervention*	34,978
#14	miscarriage* OR stillbirth* OR perinatal loss* OR perinatal grief* OR neonatal death* OR fetal death*	2229
#15	grief* OR bereavement* OR anxi* OR depress* OR post-traumatic stress* OR psychological distress* OR coping* OR adjustment* OR psychosocial wellbeing*	32,281
#16	(#13 AND #14 AND #15)	22
#17	(#16 AND #12)	5

Notes: The search strategy combined controlled vocabulary and free-text keywords covering three predefined conceptual domains: perinatal loss, psychotherapeutic/psychosocial interventions, and psychosocial outcomes. The asterisk (*) indicates a truncation symbol used to capture variations of the root term.

**Table 3:** Overview of the included reviews, intervention characteristics, and psychosocial outcomes following perinatal loss.

Authors, Year, Journal	Number of Databases and Names	Study Design, Type, and Aim	Participants/Setting/Countries	Duration and Follow-Up	Components of Intervention	Effect of Interventions
Shaohua & Shorey (2021) [37] *International Journal of Nursing Studies*	8 databases (CINAHL, Cochrane, EMBASE, ProQuest, PsycINFO, PubMed, Scopus, Web of Science)	Systematic review and meta-analysis of RCTs. Aim: Evaluate the effectiveness of psychosocial interventions on depression, anxiety, and grief after perinatal loss.	(a) N = 2065 parents (b) Hospital, community, and online settings (c) USA, UK, France, Portugal, Sweden, Iran, China, India	Follow-up ranged from 1 week to 20 months	Internet-based CBT, cognitive narrative interventions, psychological debriefing, couples counseling, telephone counseling, bereavement counseling, supportive counseling, and CBT group counseling—with a strong emphasis on therapeutic communication, emotional expression, individualized support, and active patient participation.	Reduced symptoms of depression, anxiety, and grief. Technology-assisted and individualized interventions demonstrated beneficial effects.
Dolan et al. (2022) [38] *Journal of Midwifery & Women’s Health*	5 databases (PsycINFO, CINAHL, MEDLINE, Social Science, ASSIA)	Systematic review. Aim: Compare CBT and mindfulness-based interventions for complicated perinatal grief and report participant experiences.	(a) N = 681 (b) Hospital, maternity services, home-based and online settings (c) USA, Germany, France, Japan, India	1 day to 12 weeks follow-up	CBT, mindfulness-based interventions, online CBT, mindfulness retreat, structured writing exercises, yoga-based interventions. Interventions promoted self-reflection, emotional processing, meaning-making, and patient engagement.	Reduced symptoms of grief, depression, anxiety, and post-traumatic stress. Both CBT and mindfulness-based interventions demonstrated beneficial effects.
Li et al. (2024) [22] *Depression and Anxiety*	7 databases	Systematic review and meta-analysis of RCTs. Aim: Evaluate the effectiveness of non-pharmacological interventions and identify characteristics of those that are effective.	(a) 21 RCTs (b) Hospital, community, and mixed settings (c) Multiple countries	Interventions varied across studies; short- and medium-term follow-up	Cognitive-based interventions, supportive counseling, bereavement support, psychotherapy, and psychosocial interventions. Face-to-face, individual-based interventions and ≤4 sessions were identified as optimal. Patient-centered support and social support components were prominent.	Reduced symptoms of grief, post-traumatic stress, depression, and anxiety, together with improved perceived social support. Moderate-to-high certainty of evidence.
Karaahmet & Bilgiç (2024) [39] *Omega—Journal of Death and Dying*	4 databases (PubMed/MEDLINE, Cochrane, Google Scholar, Web of Science)	Systematic review and meta-analysis. Aim: Examine the effects of psychotherapy interventions after stillbirth on grief adaptation and depression.	(a) 10 studies (b) Postpartum and bereavement care settings (c) Multiple countries	Follow-up varied across studies	Psychotherapy, mindfulness interventions, counseling, and grief-focused psychological support. Interventions emphasized supportive therapeutic relationships, emotional validation, and individualized care.	Improved grief adaptation and reduced symptoms of depression, anxiety, and stress. Psychotherapy significantly improved maternal adaptation to loss.
Huang et al. (2025) [40] *Worldviews on Evidence-Based Nursing*	7 databases	Network meta-analysis of RCTs. Aim: Compare and rank psychosocial interventions for women experiencing perinatal loss.	(a) 30 RCTs; N = 6181 women (b) Healthcare and community settings (c) Multiple countries	Follow-up varied across studies	Problem-solving therapy, bereavement support, psychosocial interventions, face-to-face and technology-assisted approaches. Focus on psychological support, patient engagement, and individualized intervention delivery.	Reduced symptoms of depression, anxiety, and grief. Problem-solving therapy ranked highest for depression and anxiety, whereas bereavement support ranked highest for grief outcomes.

Abbreviations: CBT, Cognitive Behavioral Therapy; RCT, Randomized Controlled Trial.

**Table 4:** Summary of psychosocial outcomes reported across the included reviews.

Psychosocial Outcome	Shaohua & Shorey (2021) [37]	Dolan et al. (2022) [38]	Li et al. (2024) [22]	Karaahmet & Bilgiç (2024) [39]	Huang et al. (2025) [40]	Overall Synthesis
Grief	Reduced symptoms	Reduced symptoms	Reduced symptoms	Improved adaptation	Reduced symptoms	Consistent beneficial effect (moderate certainty where assessed)
Depression	Reduced symptoms	Reduced symptoms	Reduced symptoms	Reduced symptoms	Reduced symptoms	Consistent beneficial effect (moderate certainty where assessed)
Anxiety	Reduced symptoms	Reduced symptoms	Reduced symptoms	Reduced symptoms	Reduced symptoms	Consistent beneficial effect (moderate certainty where assessed)
PTSD symptoms	—	Reduced symptoms	Reduced symptoms	—	—	Improvement reported
Stress	—	—	—	Reduced symptoms	—	Improvement reported
Perceived social support	—	—	Improved	—	—	Positive effect
Coping/adaptation	—	Improved	Improved	Improved	—	Positive effect
Psychosocial well-being	Improved	Improved	Improved	Improved	Improved	Overall improvement

Abbreviations: PTSD = Post-Traumatic Stress Disorder; Reduced symptoms = reduction in the severity of the reported psychosocial outcome following the intervention; Improved = improvement in positive psychosocial outcomes (e.g., coping, adaptation, psychosocial well-being, or social support); = outcome not specifically reported. Overall synthesis reflects the direction and consistency of findings across reviews. Where available, certainty-of-evidence assessments reported by the original review authors (e.g., GRADE) informed interpretation but were not available for all reviews.

**Table 5:** Patient-Centered Care Dimensions Identified through Secondary Interpretive Coding of the Included Systematic Reviews.

Patient-Centered Care Dimension	Shaohua & Shorey (2021) [37]	Dolan et al. (2022) [38]	Li et al. (2024) [22]	Karaahmet & Bilgiç (2024) [39]	Huang et al. (2025) [40]	Illustrative Coded Intervention Characteristics
Therapeutic communication	✓	✓	✓	✓	✓	Counseling, psychotherapy, emotional disclosure, guided discussions
Patient engagement	✓	✓	✓	✓	✓	Writing exercises, mindfulness activities, collaborative tasks
Individualized support	✓	✓	✓	✓	✓	Tailored psychological support based on individual needs
Emotional validation	✓	✓	✓	✓	U	Empathy, acceptance, and acknowledgment of grief experiences
Therapeutic relationship	✓	✓	✓	✓	U	Trust, rapport, supportive clinician–patient interactions
Meaning-making processes	✓	✓	✓	✓	—	Narrative reconstruction, reflection on loss experiences
Accessibility and continuity of care	✓	✓	✓	—	✓	Internet-based CBT, telephone counseling, online support
Technology-assisted support	✓	✓	✓	—	✓	Online interventions, digital communication platforms

Note: The patient-centered care dimensions presented in this table were identified through a predefined secondary interpretive coding framework applied to the intervention characteristics described within the included systematic reviews. Coding was performed independently by two reviewers (C.K. and T.B.) according to predefined operational definitions, as described in Section 2.4 and Section 2.5. A checkmark (✓) indicates that explicit narrative descriptions consistent with the predefined operational definition of the corresponding patient-centered care dimension were identified within the review. U indicates that reporting was insufficient or unclear to determine whether the dimension was present, whereas indicates that no relevant evidence was identified. Representative coding examples supporting each patient-centered care dimension are presented in Supplementary Table S6. The frequency with which each dimension appeared across reviews should not be interpreted as a measure of intervention effectiveness or certainty of evidence. Rather, it reflects the consistency of descriptive reporting within the included reviews. Abbreviations: CBT = Cognitive Behavioral Therapy; ✓ = patient-centered care dimension explicitly identified through the predefined secondary interpretive coding framework; U = insufficient or unclear reporting; — = no relevant evidence identified.

## Data Availability

No new primary data were created for this study. All analyzed data were derived from previously published systematic reviews and are reported in the manuscript and Supplementary Materials.

## Supplementary Materials

The following supporting information can be downloaded at: https://www.mdpi.com/article/10.3390/healthcare14142141/s1. Table S1: Search Strategy Database: Cochrane Library (from inception to 31 May 2026); Table S2: Search Strategy Database: EBSCOhost, CINAHL (from inception to 31 May 2026); Table S3: Search Strategy Database: PubMed (from inception to 31 May 2026); Table S4: Search Strategy Database: PsycINFO (from inception to 31 May 2026); Table S5: Critical Appraisal of Included Reviews Using the Joanna Briggs Institute (JBI) Critical Appraisal Checklist for Systematic Reviews and Research Syntheses; Table S6. Operational Coding Framework and Representative Examples Supporting the Secondary Interpretive Synthesis of Patient-Centered Care Dimensions; Table S7: Citation Overlap Matrix and Corrected Covered Area (CCA) Calculation across the Included Systematic Reviews.

## Abbreviations

The following abbreviations are used in this manuscript:

CBT	Cognitive Behavioral Therapy
JBI	Joanna Briggs Institute
MBI	Mindfulness-Based Intervention
PCC	Patient-Centered Care
PICOTSS	Population, Intervention, Comparator, Outcome, Time, Setting, and Study Characteristics
PRISMA	Preferred Reporting Items for Systematic Reviews and Meta-Analyses
PROSPERO	International Prospective Register of Systematic Reviews
PTSD	Post-Traumatic Stress Disorder
RCT	Randomized Controlled Trial
