# Supplementary material for: Psychotherapeutic Interventions and Psychosocial Outcomes Following Perinatal Loss: An Umbrella Review with a Patient-Centered Care Perspective
Source: Healthcare (Basel). 2026 Jul 16;14(14):2141. doi: 10.3390/healthcare14142141 (PMC13411264; doi:10.3390/healthcare14142141)
Supplement: Supplementary file 1 [file healthcare-14-02141-s001.zip › healthcare-4417071-supplementary.pdf]

Supplemental online material

Table S1. Search strategy Database: Cochrane Library 31 May 2026.

Search ID	Search term	Results
#1	MeSH descriptor: [Psychosocial Intervention] explode all trees	241
#2	MeSH descriptor: [Internet-Based Intervention] explode all trees	573
#3	MeSH descriptor: [Crisis Intervention] explode all trees	319
#4	MeSH descriptor: [Psychotherapy] explode all trees	33,845
#5	MeSH descriptor: [Anxiety] explode all trees	13,389
#6	MeSH descriptor: [Anxiety Disorders] explode all trees	9,296
#7	MeSH descriptor: [Depression] explode all trees	18,834
#8	MeSH descriptor: [Depressive Disorder] explode all trees	15,381
#9	MeSH descriptor: [Stress Disorders, Traumatic, Acute] explode all trees	58
#10	MeSH descriptor: [Abortion, Spontaneous] explode all trees	1228
#11	MeSH descriptor: [Abortion, Incomplete] explode all trees	121
#12	MeSH descriptor: [Abortion, Threatened] explode all trees	64
#13	MeSH descriptor: [Abortion, Habitual] explode all trees	388
#14	MeSH descriptor: [Fetal Death] explode all trees	549
#15	MeSH descriptor: [Fetal Resorption] explode all trees	1
#16	MeSH descriptor: [Systematic Review] explode all trees	426
Representative free-text search terms	grief, bereavement, coping*, adjustment*, psychosocial wellbeing*, depression*, anxiety*, post-traumatic stress*	Included within the free-text search strategy

Notes: The search strategy combined the controlled vocabulary presented above with additional free-text keywords covering psychotherapeutic interventions, perinatal loss, and psychosocial outcomes (including grief, bereavement, depression, anxiety, post-traumatic stress, psychological distress, coping, adjustment, and psychosocial well-being). The exact combination of controlled vocabulary and free-text terms was adapted to the indexing structure of each database while preserving the same conceptual framework across all searches.

Table S2. Search strategy Database: EBSCO host, CINAHL 31 May 2026.

Search ID	Search term	Results
S17	S13 AND S14 AND S15 AND S16 + Limiters (*Full Text; Publication Year: 2019-2025; Language: English; Age Groups: Adults (18 years and older)*)	2
S16	S12 AND S13 AND S14 AND S15	12
S15	S10 OR S11	231,709

Search ID	Search term	Results
S14	S6 OR S7 OR S8 OR S9	465,473
S13	S3 OR S4 OR S5	19,517
S12	S1 OR S2	356,317
S11	(MM "Meta Analysis") OR (MM "Meta Synthesis") OR "Meta-analysis"	114,817
S10	(MM "Systematic Review") OR "systematic review"	191,533
S9	(MM "Psychotherapeutic Processes") OR "Psychotherapeutic Processes" OR "Therapeutic Relationship" OR "Patient Engagement"	518
S8	MH "Psychosocial Intervention") OR (MH "Internet-Based Intervention") OR (MH "Crisis Intervention") OR "Intervention"	361,517
S7	(MH "Psychotherapy") OR "Psychotherapy" OR (MH "Psychotherapy, Brief") OR (MH "Psychotherapy, Group") OR (MH "Psychotherapy, Psychodynamic") OR (MH "Interpersonal Psychotherapy") OR (MH "Cognitive Therapy")	58,442
S6	(MM "Counseling") OR "Counseling"	72,053
S5	(MM "Perinatal Death")	5,193
S4	"Perinatal loss" OR "Stillbirth" OR "Perinatal death" OR "Miscarriage" OR "Perinatal grief" OR "Neonatal death"	17,923
S3	(MM "Abortion, Spontaneous") OR "Abortion, Spontaneous"	6,104
S2	(MM "Stress") OR "Stress" OR (MM "Stress Disorders, Post-Traumatic") OR (MM "Stress, Psychological")	255,248
S1	(MM "Anxiety") OR "Anxiety" OR (MM "Anxiety Disorders")	131,121
Representative free-text search terms	grief; bereavement; depression; coping; adjustment; psychological distress; psychosocial well-being; post-traumatic stress (PTSD)	Representative terms incorporated into the database-specific free-text search strategy

Notes: The search strategy combined CINAHL Subject Headings (MH/MM) with additional free-text keywords and was structured around three predefined conceptual domains: (1) psychotherapeutic and psychosocial interventions, (2) perinatal loss, and

(3) psychosocial outcomes. Representative psychosocial outcome terms included grief, bereavement, depression, anxiety, post-traumatic stress, psychological distress, coping, adjustment, and psychosocial well-being. Database-specific controlled vocabulary and free-text terms were adapted to the indexing structure of CINAHL while preserving the same conceptual framework across all four databases. Patient-centered care concepts (e.g., communication processes, therapeutic relationships, patient engagement, emotional validation, individualized support, and meaning-making) informed the secondary interpretive synthesis but were not applied as mandatory search filters or eligibility criteria.

Table S3. Search strategy Database: PubMed 31 May 2026.

	Search ID	Search string	Results
Concept Group A	1	"Anxiety"[MeSH Terms] OR "Anxiety Disorders"[MeSH Terms] OR "Anxiety"[Text Word]	19,230
	2	"Stress Disorders, Traumatic, Acute"[MeSH Terms] OR "Stress Disorders, Traumatic"[MeSH Terms] OR "Stress, Psychological"[MeSH Terms] OR "Stress Disorders, Post-Traumatic"[MeSH Terms]	19,703
	3	"Anxiety"[Text Word] OR "Anxiety Disorders"[Text Word]	32,065
	4	"Stress Disorders"[Text Word] OR "Traumatic"[Text Word] OR "Psychological Stress"[Text Word] OR "Post-Traumatic Stress"[Text Word]	25,343
Concept Group B	5	"Abortion, Spontaneous"[MeSH Terms] OR "Abortion, Incomplete"[MeSH Terms] OR "Abortion, Threatened"[MeSH Terms] OR "Abortion, Habitual"[MeSH Terms]	4,102
	6	"Fetal Death"[MeSH Terms] OR "Stillbirth"[MeSH Terms]	3,144
	7	"Abortion, Spontaneous"[Text Word] OR "Fetal Death"[Text Word] OR "Stillbirth"[Text Word] OR "Perinatal Loss"[Text Word] OR "Perinatal Grief"[Text Word] OR "Miscarriage"[Text Word] OR "Perinatal Death"[Text Word] OR "Neonatal Death"[Text Word]	7,333
	8	"abortion, spontaneous"[MeSH Terms] OR "abortion, incomplete"[MeSH Terms] OR "abortion, threatened"[MeSH Terms] OR "abortion, habitual"[MeSH Terms] OR	8,887

	Search ID	Search string	Results
Concept Group C	9	"Fetal Death"[MeSH Terms] OR "Stillbirth"[MeSH Terms] OR "abortion spontaneous"[Text Word] OR "Fetal Death"[Text Word] OR "Stillbirth"[Text Word] OR "Perinatal Loss"[Text Word] OR "Perinatal Grief"[Text Word] OR "Miscarriage"[Text Word] OR "Perinatal Death"[Text Word] OR "Neonatal Death"[Text Word] "Psychotherapy"[Mesh Terms] OR "Psychotherapy, Psychodynamic"[Mesh Terms] OR "Psychotherapy, Brief"[Mesh Terms] OR "Person-Centered Psychotherapy"[Mesh Terms] OR "Psychotherapy, Group"[Mesh Terms] OR "Cognitive Behavioral Therapy"[Mesh Terms] OR "Psychotherapy, RationalEmotive"[Mesh Terms]	22,085
	10	"Crisis Intervention"[Mesh] OR "Psychosocial Intervention"[Mesh] OR "Internet-Based Intervention"[Mesh]	846
	11	""Psychotherapy"[Text Word] OR "Psychodynamic"[Text Word] OR "Person-Centered Psychotherapy"[Text Word] OR "Group Psychotherapy"[Text Word] OR "Cognitive Behavioral Therapy"[Text Word] OR "Rational-Emotive Psychotherapy" [Text Word]	12,608
	12	"Crisis Intervention"[Text Word] OR "Psychosocial Intervention"[Text Word] OR "Internet-Based Intervention"[Text Word]	1,251
	13	"Psychotherapy"[MeSH Terms] OR "psychotherapy, psychodynamic"[MeSH Terms] OR "psychotherapy, brief"[MeSH Terms] OR "Person-Centered Psychotherapy"[MeSH Terms] OR "psychotherapy, group"[MeSH Terms] OR "Cognitive Behavioral Therapy"[MeSH Terms] OR "psychotherapy, rational emotive"[MeSH Terms] OR "Crisis Intervention"[MeSH Terms] OR "Psychosocial	24,384

	Search ID	Search string	Results
		Intervention"[MeSH Terms] OR "Internet-Based Intervention"[MeSH Terms] OR "Psychotherapy"[Text Word] OR "Psychodynamic"[Text Word] OR "Person-Centered Psychotherapy"[Text Word] OR "Group Psychotherapy"[Text Word] OR "Cognitive Behavioral Therapy"[Text Word] OR "Rational-Emotive Psychotherapy"[Text Word] OR "Crisis Intervention"[Text Word] OR "Psychosocial Intervention"[Text Word] OR "Internet-Based Intervention"[Text Word]	
Concept Group D	14	"Systematic Review" [Publication Type] OR "Systematic Reviews as Topic"[Mesh]	25,969
	15	"Meta-Analysis"[Publication Type] OR "MetaAnalysis as Topic"[Mesh]	21,565
	16	"Systematic Review" [Text Word] OR "MetaAnalysis"[Text Word] OR "Meta-synthesis" [Text Word]	46,340
	17	"Systematic Review"[Publication Type] OR "Systematic Reviews as Topic"[MeSH Terms] OR "Meta-analysis"[Publication Type] OR "Meta-analysis as Topic"[MeSH Terms] OR "Systematic Review"[Text Word] OR "Meta-Analysis"[Text Word] OR "Meta-synthesis"[Text Word]	46,559
Concept Group A AND Concept Group B AND Concept Group C AND Concept Group D	18	("stress disorders, traumatic, acute"[MeSH Terms] OR "stress disorders, traumatic"[MeSH Terms] OR "stress, psychological"[MeSH Terms] OR "stress disorders, post traumatic"[MeSH Terms] OR ("Stress Disorders"[Text Word] OR "Traumatic"[Text Word] OR "Psychological Stress"[Text Word] OR "Post Traumatic Stress"[Text Word]) OR ("Anxiety"[Text Word] OR "Anxiety Disorders"[Text Word] OR "Anxiety"[Text Word]) OR ("Anxiety"[MeSH Terms] OR "Anxiety Disorders"[MeSH Terms])) AND ("abortion, spontaneous"[MeSH Terms] OR "abortion, incomplete"[MeSH Terms] OR	6

	Search ID	Search string	Results
		"abortion, threatened"[MeSH Terms] OR "abortion, habitual"[MeSH Terms] OR ("Fetal Death"[MeSH Terms] OR "Stillbirth"[MeSH Terms]) OR ("abortion spontaneous"[Text Word] OR "Fetal Death"[Text Word] OR "Stillbirth"[Text Word] OR "Perinatal loss"[Text Word] OR "Perinatal grief" [Text Word] OR "Miscarriage"[Text Word] OR "Neonatal Death"[Text Word] OR "Perinatal death"[Text Word])) AND ("Psychotherapy"[MeSH Terms] OR "psychotherapy, psychodynamic"[MeSH Terms] OR "psychotherapy, brief"[MeSH Terms] OR "PersonCentered Psychotherapy"[MeSH Terms] OR "psychotherapy, group"[MeSH Terms] OR "Cognitive Behavioral Therapy"[MeSH Terms] OR "psychotherapy, rational emotive"[MeSH Terms] OR ("Crisis Intervention"[MeSH Terms] OR "Psychosocial Intervention"[MeSH Terms] OR "Internet-Based Intervention"[MeSH Terms]) OR ("Psychotherapy"[Text Word] OR "Psychodynamic"[Text Word] OR "Person-Centered Psychotherapy"[Text Word] OR "Group Psychotherapy"[Text Word] OR "Cognitive Behavioral Therapy"[Text Word] OR "RationalEmotive Psychotherapy"[Text Word]) OR ("Crisis Intervention"[Text Word] OR "Psychosocial Intervention"[Text Word] OR "Internet-Based Intervention"[Text Word])) AND ("Systematic Review"[Publication Type] OR "Systematic Reviews as Topic"[MeSH Terms] OR ("Meta-Analysis"[Publication Type] OR "Meta-Analysis as Topic"[MeSH Terms]) OR ("Systematic Review"[Text Word] OR "Meta-Analysis"[Text Word] OR "Meta-synthesis"[Text Word]))	
Concept Group A *AND*	19	(("stress disorders, traumatic, acute"[MeSH Terms] OR "stress disorders, traumatic"[MeSH Terms] OR "stress,	4

Search ID	Search string	Results
Concept Group B *AND* Concept Group C *AND* Concept Group D + Filters	psychological"[MeSH Terms] OR "stress disorders, post traumatic"[MeSH Terms] OR ("Stress Disorders"[Text Word] OR "Traumatic"[Text Word] OR "Psychological Stress"[Text Word] OR "PostTraumatic Stress"[Text Word]) OR ("Anxiety"[Text Word] OR "Anxiety Disorders"[Text Word] OR "Anxiety"[Text Word]) OR ("Anxiety"[MeSH Terms] OR "Anxiety Disorders"[MeSH Terms])) AND ("abortion, spontaneous"[MeSH Terms] OR "abortion, incomplete"[MeSH Terms] OR "abortion, threatened"[MeSH Terms] OR "abortion, habitual"[MeSH Terms] OR ("Fetal Death"[MeSH Terms] OR "Stillbirth"[MeSH Terms]) OR ("abortion spontaneous"[Text Word] OR "Fetal Death"[Text Word] OR "Stillbirth"[Text Word] OR "Perinatal loss"[Text Word] OR "Perinatal grief" [Text Word] OR "Miscarriage"[Text Word] OR "Perinatal death"[Text Word])) AND ("Psychotherapy"[MeSH Terms] OR "psychotherapy, psychodynamic"[MeSH Terms] OR "psychotherapy, brief"[MeSH Terms] OR "Person-Centered Psychotherapy"[MeSH Terms] OR "psychotherapy, group"[MeSH Terms] OR "Cognitive Behavioral Therapy"[MeSH Terms] OR "psychotherapy, rational emotive"[MeSH Terms] OR ("Crisis Intervention"[MeSH Terms] OR "Psychosocial Intervention"[MeSH Terms] OR "Internet-Based Intervention"[MeSH Terms]) OR ("Psychotherapy"[Text Word] OR "Psychodynamic"[Text Word] OR "Person-Centered Psychotherapy"[Text Word] OR "Group Psychotherapy"[Text Word] OR "Cognitive	

	Search ID	Search string	Results
		Behavioral Therapy"[Text Word] OR "Rational Emotive Psychotherapy"[Text Word]) OR ("Crisis Intervention"[Text Word] OR "Psychosocial Intervention"[Text Word] OR "Internet-Based Intervention"[Text Word])) AND ("Systematic Review"[Publication Type] OR "Systematic Reviews as Topic"[MeSH Terms] OR ("Meta-Analysis"[Publication Type] OR "Meta-Analysis as Topic"[MeSH Terms]) OR ("Systematic Review"[Text Word] OR "Meta-Analysis"[Text Word] OR "Meta-synthesis"[Text Word]))) AND ((y_5[Filter]) AND (fft[Filter]) AND (english[Filter]))	
Representative free-text psychosocial outcome terms	Grief; bereavement; depression; coping; adjustment; psychological distress; psychosocial well-being; post-traumatic stress (PTSD)	Representative terms incorporated into the database-specific free-text search strategy	Representative free-text psychosocial outcome terms

Filters: Humans; Adults (18 years and older); English language; Full-text articles; Publication years 2019–2025.

Notes: The PubMed search strategy combined MeSH terms with additional free-text keywords and was structured around four predefined conceptual domains: (1) psychosocial outcomes, (2) perinatal loss, (3) psychotherapeutic and psychosocial interventions, and (4) systematic reviews/meta-analyses. Representative psychosocial outcome terms are presented above for clarity and to illustrate the breadth of the outcome-related terminology used during database searching**.** These included grief, bereavement, depression, anxiety, post-traumatic stress, psychological distress, coping, adjustment, and psychosocial well-being. Database-specific controlled vocabulary and free-text keywords were adapted to the indexing structure of PubMed while preserving the same conceptual framework across all databases. Patient-centered care concepts (e.g., communication processes, therapeutic relationships, patient engagement, emotional validation, individualized support, and meaning-making) informed the secondary interpretive synthesis but were not applied as mandatory search filters or eligibility criteria.

Table S4. Search strategy Database: PsycINFO 31 May 2026.

Search ID	Search term	Results
S18	S13 AND S14 AND S15 AND S16+ Limiters *(Full Text; Publication Year: 2019-2025; Language: English)*	1
S17	S13 AND S14 AND S15 AND S16	2
S16	S11 OR S12	77,988
S15	S6 OR S7 OR S8 OR S9 OR S10	721,956
S14	S4 OR S5	2,534
S13	S1 OR S2 OR S3	569,198
S12	MM "Meta-analysis" OR "Meta-analysis" OR "Meta Synthesis"	45,267
S11	MM "Systematic Review" OR "Systematic Review"	50,738
S10	MM "Psychotherapeutic Processes" OR "Psychotherapeutic Processes" OR MM "Psychotherapeutic Techniques"	53,523
S9	MM "Crisis Intervention" OR "Crisis Intervention"	12,201
S8	MM "Psychotherapy" OR "Psychotherapy"	259,426
S7	MM "Counseling" OR "Counseling" OR MM "Counseling Psychology"	239,936
S6	MM "Intervention" OR "Intervention"	361,397
S5	"Perinatal loss" OR "Stillbirth" OR "Perinatal death" OR "Neonatal death" OR "Miscarriage" OR "Perinatal grief"	2,355
S4	MM "Spontaneous Abortion" OR "Spontaneous Abortion"	1,152
S3	MM "Posttraumatic Stress" OR "Posttraumatic Stress" OR MM "Posttraumatic Stress Disorder"	52,083
S2	MM "Stress" OR "Stress" OR MM "Stress and Trauma Related Disorders"	332,410
S1	MM "Anxiety" OR DE "Anxiety Disorders" OR MM "Anxiety Management" OR "Anxiety"	322,561
Representative free-text psychosocial outcome terms	Grief; bereavement; depression; coping; adjustment; psychological distress; psychosocial well-being; post-traumatic stress (PTSD)	Representative terms incorporated into the database-specific free-text search strategy

Notes: The PsycINFO search strategy combined controlled vocabulary (APA Thesaurus descriptors) with additional free-text keywords and was structured around four predefined conceptual domains: (1) psychosocial outcomes, (2) perinatal loss, (3) psychotherapeutic and psychosocial interventions, and (4) systematic reviews/meta-analyses. Representative psychosocial outcome terms are presented above for clarity and to illustrate the breadth of the outcome-related terminology used during database

searching. These included grief, bereavement, depression, anxiety, post-traumatic stress, psychological distress, coping, adjustment, and psychosocial well-being. Database-specific controlled vocabulary and free-text keywords were adapted to the indexing structure of PsycINFO while preserving the same conceptual framework across all databases. Patient-centered care concepts (e.g., communication processes, therapeutic relationships, patient engagement, emotional validation, individualized support, and meaning-making) informed the secondary interpretive synthesis but were not applied as mandatory search filters or eligibility criteria.

Table S5. Critical appraisal of included reviews using the JBI Critical Appraisal Checklist for Systematic Reviews and Research Syntheses.

**Review (*n* =5)**												
**Authors**	**1**	**2**	**3**	**4**	**5**	**6**	**7**	**8**	**9**	**10**	**11**	**Comments**
**Shaohua & Shorey (2021) [37]**	Y	U	Y	Y	Y	Y	Y	Y	Y	Y	Y	The PICO framework was not explicitly reported.
**Dolan et al. (2022) [38]**	Y	Y	Y	Y	Y	Y	Y	Y	U	Y	Y	Publication bias assessment was not clearly reported.
**Li et al. (2024) [22]**	Y	Y	Y	Y	Y	Y	Y	Y	Y	Y	Y	GRADE certainty-of-evidence assessment performed; moderate-to-high certainty reported for several outcomes.
**Karaahmet & Bilgiç (2024) [39]**	Y	Y	Y	Y	Y	Y	Y	Y	Y	Y	Y	Risk of bias assessed using ROB-2 and ROBINS-I.
**Huang et al. (2025) [40]**	Y	Y	Y	Y	Y	Y	Y	Y	Y	Y	Y	GRADE/CINeMA certainty assessment incorporated within the network meta-analysis.
Y=Yes, N=No, U=Unclear, NA= Not applicable												

1. Is the review question clearly and explicitly stated?
2. Are the inclusion criteria appropriate for the review question?
3. Was the search strategy appropriate?
4. Were the sources and resources used to search for studies adequate?
5. Were the criteria for appraising studies appropriate?
6. Was a critical appraisal conducted by two or more reviewers independently?
7. Were there methods to minimize errors in data extraction?
8. Were the methods used to combine studies appropriate?
9. Was the likelihood of publication bias assessed?
10. Were recommendations for policy and/or practice supported by the reported data?
11. Were the specific directives for new research appropriate?

Table S6. Operational Coding Framework and Representative Examples Supporting the Secondary Interpretive Synthesis of Patient-Centered Care Dimensions.

Patient-centered care dimension	Operational definition	Representative examples extracted from the included systematic reviews*	Example review(s)
**Therapeutic communication**	Structured therapeutic interactions facilitating emotional expression, discussion of loss experiences, active listening, counselling, or guided communication between healthcare professionals and participants.	Bereavement counselling; supportive counselling; psychotherapy sessions; guided emotional disclosure; therapist-led discussions.	Shaohua & Shorey (2021) [37]; Karaahmet & Bilgiç (2024) [39]; Li et al. (2024) [22].
**Patient engagement**	Active participation of participants in therapeutic activities requiring reflection, collaboration, or behavioral involvement.	Structured writing exercises; mindfulness practices; homework activities; collaborative therapeutic tasks; self-monitoring exercises.	Dolan et al. (2022) [38]; Li et al. (2024) [22]; Shaohua & Shorey (2021) [37].
**Individualized support**	Adaptation of intervention content according to participants' emotional needs, grief experiences,	Tailored psychological support; individualized counselling; personalized intervention planning;	Li et al. (2024) [22]; Karaahmet & Bilgiç (2024) [39]; Huang et

	or personal circumstances.	needs-based psychological care.	al. (2025) [40].
**Emotional validation**	Explicit acknowledgement, acceptance, normalization, or empathic response to participants' emotional reactions following perinatal loss.	Validation of grief responses; empathic listening; acceptance of emotional experiences; supportive acknowledgement of loss.	Shaohua & Shorey (2021) [37]; Karaahmet & Bilgiç (2024) [39].
**Therapeutic relationship**	Descriptions of trust, rapport, therapeutic alliance, continuity of professional support, or supportive clinician–patient interactions.	Therapeutic alliance; trusting clinician–patient relationship; supportive interaction; continuity of counselling.	Shaohua & Shorey (2021) [37]; Li et al. (2024) [22]; Karaahmet & Bilgiç (2024) [39].
**Meaning-making processes**	Intervention components encouraging reflection, reinterpretation of the loss experience, or construction of personal meaning.	Narrative reconstruction; storytelling; reflective writing; meaning-oriented discussions; expressive writing.	Dolan et al. (2022) [38]; Shaohua & Shorey (2021) [37].
**Accessibility and continuity of care**	Intervention characteristics facilitating ongoing psychological support or improving access to care.	Telephone counselling; follow-up contacts; internet-based CBT; remote psychological support; online follow-up.	Shaohua & Shorey (2021) [37]; Huang et al. (2025) [40].

Note: Representative examples summarize intervention characteristics explicitly reported in the included systematic reviews and do not represent direct quotations or independent coding of the original randomized controlled trials. The examples

are illustrative rather than exhaustive and do not represent direct quotations from the original randomized controlled trials. Coding was performed independently by two reviewers (C.K. and T.B.) using the operational definitions presented in Sections 2.4 and 2.5 of the main manuscript**.**

Table S7. Citation overlap matrix of the primary studies included in the five systematic reviews.

**Primary study**	**Shaohua et al.**	**Dolan et al.**	**Li et al.**	**Karaahmet et al.**	**Huang et al.**
Bennett et al., 2012	✓	✓		✓	
Forrest et al., 1982				✓	
Haghighi et al., 2022				✓	✓
Huberty et al., 2020	✓	✓		✓	✓
Johnson et al., 2015	✓			✓	
Johnson et al., 2016	✓			✓	
Kersting et al., 2011	✓	✓	✓	✓	✓
Kersting et al., 2013	✓		✓	✓	✓
Kersting et al., 2017	✓	✓	✓		✓
Lake et al., 1987				✓	
Nakano et al., 2013	✓	✓			
Nasrollahi et al., 2022				✓	✓
Navidian et al., 2017			✓	✓	

Navidian et al., 2018	✓		✓	✓	✓
Roberts & Montgomery, 2016	✓	✓	✓		✓
Séjourné et al., 2010	✓	✓			
Simpson et al., 2015				✓	
Thieleman & Cacciatore, 2020	✓	✓	✓		✓

Corrected Covered Area (CCA) calculation

**Item**	**Value**
Number of included systematic reviews (c)	5
Number of unique primary studies (r)	18
Total study occurrences across reviews (N)	49
Corrected Covered Area (CCA)	43.1%
Interpretation	Very high overlap

Abbreviations: ✓ = primary study included in the respective systematic review.
